# What Are We Doing Wrong When Athletes Report Higher Levels of Fatigue From Traveling Than From Training or Competition?

**DOI:** 10.3389/fpsyg.2020.00194

**Published:** 2020-02-21

**Authors:** Julio Calleja-Gonzalez, Diego Marques-Jimenez, Margaret Jones, Thomas Huyghe, Fernando Navarro, Anne Delextrat, Igor Jukic, Sergej M. Ostojic, Jaime E. Sampaio, Xavi Schelling, Pedro E. Alcaraz, Fernando Sanchez-Bañuelos, Xavier Leibar, Juan Mielgo-Ayuso, Nicolas Terrados

**Affiliations:** ^1^Department of Physical Activity and Sports, University of the Basque Country, Bilbao, Spain; ^2^Deportivo Alavés, Academy Department, Álava, Spain; ^3^Catholic University San Antonio of Murcia, Murcia, Spain; ^4^Sport Training Lab, Faculty of Sport Sciences, University of Castilla—La Mancha, Toledo, Spain; ^5^Department of Sport and Health Sciences, Oxford Brookes University, Oxford, United Kingdom; ^6^Faculty of Kinesiology, University of Zagreb, Zagreb, Croatia; ^7^Faculty of Sport and Physical Education, University of Novi Sad, Novi Sad, Serbia; ^8^Research Center in Sports Sciences, Health Sciences and Human Development (CIDESD), University of Trás-os-Montes e Alto Douro (UTAD), Vila Real, Portugal; ^9^Institute of Sport, Exercise and Active Living, College of Sport and Exercise Science, Victoria University, Melbourne, VIC, Australia; ^10^Research Center for High Performance Sport, Catholic University of Murcia, Murcia, Spain; ^11^Faculty of Sport Sciences, Catholic University of Murcia, Murcia, Spain; ^12^Spanish Olympic Committee, Madrid, Spain; ^13^Department of Biochemistry Molecular Biology and Physiology, Faculty of Health Sciences, Campus de Soria, University of Valladolid, Soria, Spain; ^14^Regional Unit of Sports Medicine, Aviles and Health Research Institute of the Principality of Asturias (ISPA), Oviedo, Spain

**Keywords:** fatigue, competition, sport, TRIP, training, travel, recovery

Performance at the elite level in running-based team sports requires outlining the cyclical nature in which physiological and biomechanical loads lead to adaptation of the biological system as a whole (Vanrenterghem et al., 2017). Very commonly, there are congested fixture periods that seem to have no effect on physical activity, technical performance, and injury incidence (Dellal et al., 2015) injury rates or patterns (Carling et al., 2016), but do seem to decrease tactical performance, as measured by levels of movement synchronization (Folgado et al., 2015).

A very high traveling frequency is required to compete in elite professional sport. For example, the National Basketball Association's regular season consists of 82 games (41 home, 41 away) played over a 6-month period (Sampaio et al., 2015). This can have consequences for both physiological and psychological status and has the potential to impair performance, as seen in common anecdotal elite basketball player reports stating: “*I want to sleep,”* “*I didn't sleep enough,” “I slept poorly,” “I get tired of traveling”; “I prefer to sleep at home even if it means getting home late.”*

The sentiments and feelings like the aforementioned may clearly affect the balance between happiness and wellness (Calleja-Gonzalez et al., 2018). In that way, coaches focus on respecting, valuing, involving, engaging in dialogue with, listening to, and supporting players, as well as treating them as human beings, giving them the confidence and feelings of responsibility to try (Barker-Ruchti et al., 2014). There is a clear need for more research in this area, although some advances were already made by examining empathy using qualitative methods and identifying factors of empathy between athletes and coaches (David and Larson, 2018). Furthermore, a period of constructive reflection considering the relationship between performance analysis and recovery is strongly recommended (Calleja-González et al., 2018). Thus, there is a gap between research and reality (Buchheit, 2017), because players express that they are more fatigued from traveling than from training or competition, which is the focus of this letter.

A shift in the approach to sports performance research seems to be necessary. For example, sleep quality and quantity (Gupta et al., 2017), burden associated to traveling (Fowler et al., 2014), chronobiological disturbance (Drust et al., 2005) are often cited as limiting factors of performance in high level sport, and their impact should be considered and assessed. Further, the additive effect or the means by which one factor influence another should be taken into account (Tobias et al., 2013).

Elite athletes are exposed to substantial training loads (Soligard et al., 2016), however, that is only a (small) part of the key determinants of performance. Current trends in expertise describe the concept as a dynamically varying relationship captured by the constraints of the environment and those of the performer of a task (McGuckian et al., 2018). Using this approach, the context is key and should not be detached from the content, thus, the guidelines for designing and implementation of a training program will benefit from incorporating environmental information, integrated periodization, mental performance, skill acquisition, or nutrition (Mujika et al., 2018). In addition, using the aforementioned methods in combination with athlete monitoring of training, competition and psychological load, and pooled with assessments of recovery, well-being, and illness (Schwellnus et al., 2016). It may enable the achievement of enhanced performance levels.

Since extended traveling is common in elite sport (Flatt et al., 2019), it is recommended that coaches and applied sports scientists consider the following key points in order to minimize injury risk, enhance recovery, optimize performance and reduce the effect of traveling and sleep disturbance on performance with elite team sports players (Vitale et al., 2019):

- Monitoring external training load (before, during and after competition) using tracking systems (Fox et al., 2017) with the least possible invasion.
- Monitor Internal responses using heart rate measures and biomarkers in blood, saliva, and/or urine before, during and after competition (Halson, 2014).
- Monitor daily sleep quality, sleep duration, and player well-being to inform same day adjustments to training and competition workload (Fox et al., 2019).
- Arrive early to competition destination in order to include sufficient time on-site to recover from traveling and adjust to new time-zones, altitudes, climates, and environments (Lastella et al., 2019).
- Avoid environmental changes because changing physical sleep environments may increase susceptibility to altered sleep responses, which may negatively affect performance (Pitchford et al., 2017).
- Develop and apply consistent strategies (pre, during and post-traveling) that may help prevent or ease jet lag (Fowler et al., 2014).
- Develop and apply an *ad-hoc* nutrition plan for traveling (Halson et al., 2019).

Stress on the body is probably cumulative (Issurin, 2009). Therefore, the development of new variables, such as ratios, that might relate player's fatigue, training demands, match performance, environmental conditions, at home or away, could be an interesting open window to explore. Further, the creation and validation of a travel fatigue scale would enhance an understanding of the traveling effect. Also, a scale of mental fatigue (Russell et al., 2019) that informs about the stress derived from training, competition and environmental stress would be most useful.

With the increasing popularity of sport, number of contests, and travel demands on the rise, the importance of athlete load monitoring in combination with nutritional programming, implementation of recovery methods, and proper sleep practices cannot be underestimated. Taking these steps will make for a more effective travel experience and support athlete health and playing career longevity. In the same way, rationalizing the use of measurement instruments and procedures seems also a need, as anecdotally suggests that “strict data-led regimes undermine trust and stifle creativity, shackling a player's natural empathy with the game,” thus, “it is vital that those who oversee performance in elite sport consider the consequences on players of such intense surveillance.”

Finally, novel scientific studies examining the impact of air traveling direction, flight time, flight duration, average flight altitude (above sea level), frequency and magnitude of height changes during flight, air cabin conditions, oxygen saturation levels, and athlete chronotype are warranted to help painting a clearer picture on how different stressors impact wellness and performance due to traveling. Athlete monitoring tools may help to understand how each of the above-mentioned variables play a role on the accumulation of both acute and chronic fatigue in elite athletes. However, common wearable technologies and test procedures may still present a burden in terms of practicality, time efficiency, reliability, and/or validity. Therefore, novel easy-to-use methodologies such as the critical fusion threshold (Clemente-Suarez and Diaz-Manzano, 2019) and Ruler Test (Eckner et al., 2015; van Schooten et al., 2019) may facilitate our ability to measure and monitor the rigors of traveling on a daily basis, specifically pertaining to its consequences on the central nervous system and psychophysics in elite athletes. However, further research and clinical trials are needed to validate its applicability. Additional topics should be considered in future researches and practical solutions such as:

- Bus/plane traveling (seats ergonomic, number of disposable seats in bus/plane) (Menegon et al., 2019).
- Seating positions/dangerous seating positions (players education and control).
- Muscle activation during traveling (Smulders et al., 2019).
- Intellectual activity during traveling.
- Problem with sleep medicaments (hypotonic effects) (DeKosky and Williamson, 2020).
- Sleep banking between travels and games (Roy and Forest, 2018).
- Designing individual players traveling profile.
- Plane/bus vibration effect on athlete's bodies (Blake et al., 2018).
- Plane/bus engine noise stressor effect (Hede, 2017).

## Author Contributions

JC-G: original idea and conception and design. DM-J: analysis and interpretation. MJ and JS: critically reviewed. TH, FN, AD, IJ: drafting the paper. SO, PA, and NT: final approval. XS and JM-A: approved the final version. FS-B and XL: interpretation. All authors listed have made a substantial, direct and intellectual contribution to the work, and approved it for publication.

### Conflict of Interest

DM-J was employed by company Deportivo Alavés, Spain. The remaining authors declare that the research was conducted in the absence of any commercial or financial relationships that could be construed as a potential conflict of interest.
